# Mini-Tablets: A Valid Strategy to Combine Efficacy and Safety in Pediatrics

**DOI:** 10.3390/ph15010108

**Published:** 2022-01-17

**Authors:** Guendalina Zuccari, Silvana Alfei, Danilo Marimpietri, Valentina Iurilli, Paola Barabino, Leonardo Marchitto

**Affiliations:** 1 Department of Pharmacy, University of Genoa, Viale Cembrano, 16148 Genoa, Italy; alfei@difar.unige.it; 2 Stem Cell Laboratory and Cell Therapy Center, IRCCS Istituto Giannina Gaslini, Via Gerolamo Gaslini 5, 16147 Genoa, Italy; danilomarimpietri@gaslini.org; 3 Pharmacy, IRCCS Istituto Giannina Gaslini, Via Gerolamo Gaslini 5, 16147 Genoa, Italy; valentinaiurilli@gaslini.org (V.I.); paolabarabino@gaslini.org (P.B.); 4 Department of Sciences for the Quality of Life, University of Bologna, Corso D’Augusto 237, 47921 Rimini, Italy; leonardo.marchitto@unibo.it

**Keywords:** pediatric formulations, mini-tablets, European Paediatric Regulation, excipients, medicines for children, solid dosage forms, flexible dose

## Abstract

In the treatment of pediatric diseases, mass-produced dosage forms are often not suitable for children. Commercially available medicines are commonly manipulated and mixed with food by caregivers at home, or extemporaneous medications are routinely compounded in the hospital pharmacies to treat hospitalized children. Despite considerable efforts by regulatory agencies, the pediatric population is still exposed to questionable and potentially harmful practices. When designing medicines for children, the ability to fine-tune the dosage while ensuring the safety of the ingredients is of paramount importance. For these purposes solid formulations may represent a valid alternative to liquid formulations for their simpler formula and more stability, and, to overcome the problem of swelling ability, mini-tablets could be a practicable option. This review deals with the different approaches that may be applied to develop mini-tablets intended for pediatrics with a focus on the safety of excipients. Alongside the conventional method of compression, 3D printing appeared particularly appealing, as it allows to reduce the number of ingredients and to avoid both the mixing of powders and intermediate steps such as granulation. Therefore, this technique could be well adaptable to the daily galenic preparations of a hospital pharmacy, thus leading to a reduction of the common practice of off-label preparations.

## 1. Introduction

People respond very differently to medications depending on their weight, age, inborn capability to metabolize drugs, the individual characteristics of the disease, or the presence of co-morbidities. The well-known assumption that “one size fits all” has clearly revealed all its limitations and become questionable. Notably, among humans, pediatric patients represent an inhomogeneous and very vulnerable community, thus further complicating the situation [1]. In this context, medicinal treatments can be extremely challenging, since children differ from adults in their ability to swallow dosage forms, affording formulation-related toxicity, and in taste preferences. Moreover, taking into consideration that the pharmacokinetic and pharmacodynamic profile of a drug varies extensively according to the child’s age, pediatric medicines must change consistently in their size along with the child’s growth, thus requiring flexible doses and variable administration routes. A single product may not be feasible for all the subset-population, which comprises preterm newborn infants, term newborn infants (0–8 days), infants and toddlers (1 month–2 years), pre-school children (2–5 years), school children (6–11 years), and adolescents (12–16/18 years) [2]. Collectively, among the overall patient population, the pediatric one requires the highest dose flexibility.

The most available marketed drug formulations are not intended specifically for pediatrics; consequently, pharmacists in hospitals or caregivers at home must manipulate them to obtain the appropriate dose. Particularly, they crush tablets and blend them into food, in a way that is not reported in the summary of the product characteristics (SmPC) without considering the effects that a change in pH, viscosity, fat, and sugar content, may exert on the drug bioavailability. This questionable practice of off-label drug use is not subjected to the proper degree of regulation and quality control of a licensed medicinal product, and it could even negatively affect the outcome of the disease [3]. 

Based on the aforementioned considerations, advances in the development of medicines specifically intended for use in pediatrics are urgently needed. The preparation of such dosage forms should be realized both in pharmaceutical industries or in community and hospital pharmacies. Recent innovations, such as 3D printing technologies, may facilitate these procedures, but, unfortunately, the regulatory guidelines and the pharmacopoeias still lack appropriate methods for technological quality control, and a global harmonization is needed. In this scenario, since mini tablets (MTs) represent one of the most promising dosage forms intended for pediatrics, in the herein review, we have provided updated information concerning the current state of the art of this specific dosage form. Firstly, we have focused on describing the regulatory aspects to highlight the European Medicines Agency (EMA) efforts in creating opportunities and initiatives for the industry stakeholders, with a special emphasis on the main ingredients and on the manufacturing processes up to today for producing MTs. Particularly, after systematic research of the literature, the available technologies, formulations, and marketed MT products have been critically evaluated and herein reported. To originally complete our review article and for better identification of unmet pediatric needs, the experience of the hospital pharmacy at the G. Gaslini Children’s Hospital was also included. 

## 2. European Regulatory Aspects

Over the last few years, to avoid the common practice of dividing or crushing tablets and their mixing with food or beverage, starting from the first-round table of experts organized in 1997, the European regulatory authorities have directed massively their efforts towards the development of pediatric formulations [4]. Moreover, in that context, it was established that there was a need to strengthen the legislation, particularly by introducing a system of incentives. Aimed at facilitating the organization of safe and ethical pediatric clinical trials internationally, the International Council for Harmonisation of Technical Requirements for Pharmaceuticals for Human Use (ICH) contributed to pediatric drug development through the E11 guideline “Clinical Investigation of Medicinal Products in the Pediatric Population” released in 2000, which became the European guideline “Note for guidance on clinical investigation of medicinal products in the paediatric population” in 2001 [5]. Its purpose was to increase available information on medicines for children avoiding unnecessary studies and without delaying the authorizations for medicines intended for adults. The ICH E11 guidance provided information on when pharmacokinetic and bioequivalence studies have to be performed in the paediatric population [5]. It is stated that “*when a medicinal product is to be used in the paediatric population for the same indication(s) as those studied and approved in adults, the disease process is similar in adults and paediatric patients, and the outcome of therapy is likely to be comparable, extrapolation from adult efficacy data may be appropriate*”. The guidance also proposed as acceptable the extrapolation from older to younger pediatric patients when disease progression is similar. Further specific interventions concerning children protection in clinical trials were included in the Directive (2001/20/EC) on Good Clinical Practice for Clinical Trials which came fully into force in May 2004 [6]. 

To address the problem of the use of unlicensed formulations, the EMA released a consultation paper titled “Better medicines for children—proposed regulatory actions on pediatric medicinal products” (2002) [7]. Subsequently, aiming at sharing helpful and available information relevant to the issue, all the comments received were included and summarized by the Committee for Medicinal Products for Human Use (CHMP) in the “Reflection paper: formulations of choice for the paediatric population” [2]. 

In January 2007, the European Paediatric Regulation “Better medicines for children” entered into force with the scope to greatly incentivize the number of authorized pediatric medicinal products [8]. Indeed, due to this legislation, it is no longer possible to apply for new drugs or for patent authorization extension without taking children into account. The Regulation enforces children to be included early during drug development. In particular, the companies are obliged to develop a pediatric investigation plan (PIP) and submit it at the end of clinical phase I, despite the understanding of the new drug’s effect is just emerging. Companies are allowed to apply for drug approval at the EMA only after having obtained the PIP agreement by the Paediatric Committee (PDCO). Moreover, according to the EMA, “*A PIP requirement also applies when a marketing-authorization holder wants to add a new indication, pharmaceutical form or route of administration for a medicine that is already authorized and covered by intellectual property rights*”. This has strongly contributed to a remarkable increase in the expansion of the production of drugs for the pediatric population, thus bringing the pediatric drug development more in line with that of adults. Unlike patent-protected products, the preparation and submission of a PIP for an off-patent drug is optional. In this case, applicants may apply for a paediatric use marketing authorization (PUMA) and the product necessarily must be intended for pediatric use, but it will benefit a total of 10 years of data protection.

**Figure 1:**
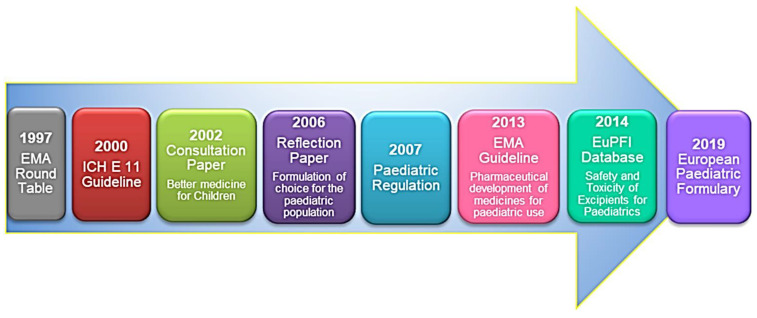
The European path of pediatric formulations.

However, following the EMA awareness concerning the paucity and disorganization of the available knowledge about pediatric formulations, several research consortia were promoted, such as the European Paediatric Formulation Initiative (EuPFI) and the European Study of Neonatal Excipient Exposure (ESNEE). At the same time, a guideline on the pharmaceutical development of medicines for pediatric use was launched by the CHMP in 2013 [9]. The guideline requires companies to consider several aspects in the pharmaceutical design of a pediatric medicinal product. Considerations such as the route of administration, dosage form, excipient composition, administration device, patient acceptability, dosing frequency are the main topics therein discussed. Recently, in December 2019, the European Committee on Pharmaceuticals and Pharmaceutical Care (CD-P-PH) and the European Pharmacopoeia Commission launched the European Paediatric Formulary on a dedicated online platform free of charge [10]. For the first time, formulations of appropriate quality from all around Europe were collected, thus allowing pharmacists and clinicians to prepare pediatric treatments when any licensed alternative is not available (Figure 1).

Currently, thanks to EMA guidance, the number of approved drugs with specific efficacy and safety data in labeling for pediatric populations has increased. However, in many cases, there is still a large gap (about 7–10 years) between the initial adult approval and the inclusion of pediatric-specific information in product labeling. Recently, the addendum (R1) to the ICH guideline E11 focused on the factors to be considered to speed up the program process for the development of pediatric medicinal products, the timing of pediatric pharmacokinetic studies, and of pediatric drug production [11]. Another advance towards these facilitations is represented by the ICH E 11A concept paper, which proposes strategies for harmonizing the methodologies and for incorporating pediatric extrapolations into drug development plans, thus limiting the number of children required for enrolment in clinical trials. “*Pediatric extrapolation*” is defined as an approach based on the assumption that the expected response to a medicinal product would be sufficiently similar in the pediatric community and in the reference population (adult or other pediatric) [12].

**Figure 2:**
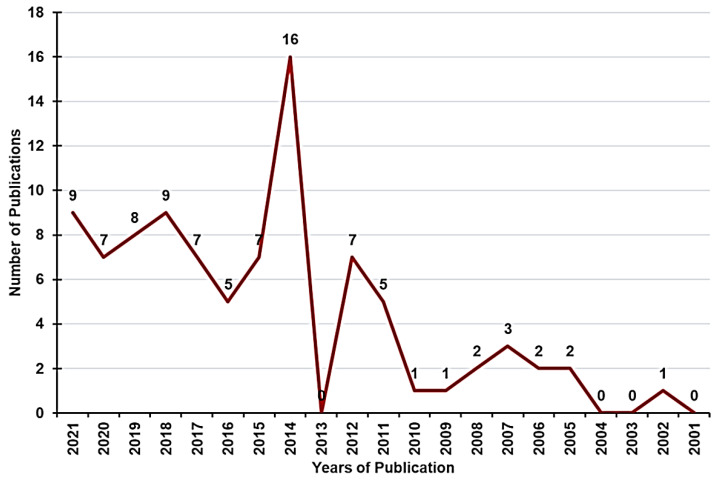
Number of publications per year of the last 20 years according to Scopus.

Nowadays, because of the above-mentioned initiatives, many medicines for pediatrics are under development, as confirmed by the recent 10-year-report on the experience acquired following the application of the pediatric regulation [13]. Several new PIPs were submitted and an increase in clinical studies was noted; thus the trend of disregarding pediatric patients has been reversed. To give readers an idea of the growing scientific interest in the development of medicines and drug formulations for pediatrics in the last 20 years, we carried out a survey of the number of works published year-by-year from 2001 to today, conducting a search by keywords in Scopus (Figure 2).

While the articles reporting studies for the development of pediatrics formulation was low up to the year 2011 in the last decade, it has grown considerably. However, pediatric medicines are still mostly linked to adult drug development, and investments in products specifically for children or rare children’s diseases remain very limited. Notably, the regulation failed in stimulating research in off-patent substances and in helping to transform well-known off-label use into authorized use. Indeed, few are PUMAs for off-patent substances, as they are still not particularly appealing for sponsors, although the warranty for the manufacturer of ten years is for marketing protection [13]. Therefore, the variety of generic formulations available on the market is still too often manipulated and administered to children through unlicensed and off-label use.

### Enalapril: From an Off-Label to a Licensed Use via Oro-Dispersible Mini-Tablet Preparation

The efforts of the European Union in supporting the transformation from off-label to fully licensed use of medicines for pediatrics led to the creation of the “Labeling of Enalapril from Neonates to Adolescents” (LENA) project, which included seven European countries. It aimed at clinically investigating and developing an enalapril medicinal product, easily and safely administrable to all pediatric subpopulations [14]. Concurrently, the collected data served to generate sufficient information for obtaining the approval of a PUMA for the product. Particularly, enalapril is a prodrug, since in vivo it is hydrolyzed to the more potent ACE inhibitor enalaprilat. After careful consideration, the most suitable formulation appeared to be MTs. The main reasons at the basis of this decision were the drug’s intrinsic instability in water and its susceptibility to degradation under heating. Furthermore, the prolonged use of solutions rich in preservatives without available data on their long-term safety, together with the observed dental erosion associated with the intake of the enalapril acidic solutions, led the investigators to opt for oro-dispersible MTs. The MTs were prepared by direct compression. Among the fillers suitable for oro-dispersible MTs, sugar alcohols seemed the best choice as they may be responsible only for a possible osmotic laxative effect, due to their poor absorption in the gastrointestinal tract. Luckily, the negligible amounts used in MTs manufacturing are surely under the laxation threshold. Additional flavoring agents and sweeteners were considered unnecessary, as sugars were already present as fillers. Regarding the available coloring agents, the LENA team opted for the iron-oxide colorants, as they are usually regarded as safer. Finally, the required dose of API was identified thanks to a close collaboration with clinicians. Since the enalapril dosing is gradually increased until the final maintenance dose is reached, more than one MT strength was developed (0.25 and 1 mg). In preparing such doses, good flowability and blend homogeneity were particularly important to warrant constant die filling. The MTs formula contained enalapril maleate, Ludiflash^®^, sodium stearyl fumarate, and yellow iron oxide (for the 1 mg MTs only). Two hundred MTs were packaged in multi-dose containers with a child-proof closure system, including a desiccant in the cap to prevent water uptake from air moisture. On request of the PDCO, a method for administering an extemporaneous dispersion of one MT or of five MTs containing 0.025 mg or 1.25 mg of enalapril, respectively, was successfully developed using an oral dosing syringe. The results of the disintegration test revealed that MTs disintegrated faster in tap water (8 s), whereas milk retarded the disintegration up to five times longer, suggesting that the MTs dispersion in milk should be avoided. Additionally, according to a PDCO request, the feasibility of the nasogastric administration was proven and the compatibility with materials such as silicone, PUR, and PVC was assessed, to evaluate the adsorption of the API onto the nasogastric tube [15]. Pharmacokinetic studies were performed as part of an open-label, multicenter clinical trial in children affected by heart failure. The generated clinical data will enable the transformation of enalapril in pediatrics from off-label to fully licensed use [16].

## 3. Appropriate Excipients for Pediatrics

In principle, almost all drug formulations contain excipients that have been used for many years and are considered to have a generally regarded as safe (GRAS) status. They are described in monographs in various pharmacopeias and released with certificates of analyses, performed according to monograph test methods, that warrant their quality. In any case, the specifications embedded in the monographs are intended to cover use in adults and not in children. Consequently, although significant differences in pharmacokinetics and pharmacodynamics exist between the two patient populations, it is common practice to assume that excipients, which did not cause adverse reactions in adults, are safe also in neonates and/or children. In recent years, awareness that some excipients are less well tolerated in children, especially in neonates whose physiological systems are still undergoing development, has become known for the intervention of the regulatory authorities. In fact, some neonates may not be able to clear an excipient with the same rate as adults, as in cases of phenylketonuria. Therefore, not only the choice of the most suitable formulation but also the selection of excipients represent key factors in the development of adequate pediatric dosage forms. In the EMA guideline, it is stated how the selection of a safe excipient can be performed [9]. In projecting a new formulation, the EMA guideline suggests that a certain excipient should be chosen by drawing on the sources listed below in hierarchical order. Commission, ICH, and EMA guidelines

- CHMP scientific opinions
- Already authorized in pediatric medicines with known quantitative composition
- Included in the European Food Legislation or Included in EFSA opinions
- Other sources such as the expert committee on food additives (JECFA), indexed
- Literature, or in-house scientific evidence

However, the opinions regarding food rarely comprise neonates [17], and if the information about safety is not available, additional costs to sustain animal and clinical toxicological studies are needed. As a result, the excipients used are not new in most cases. Moreover, the EMA guideline suggests that, despite the use of a novel excipient is important for pharmaceutical innovation, only a large-scale employment can provide more reliable data on its safety. Aiming at addressing some of the issues concerning the substances to be considered safe for developing pediatrics formulations, European and US Pediatric Formulations Initiatives (EU-US PFI) are working to collect from disparate resources available data, and to gather them under the umbrella of the Safety and Toxicity of Excipients for Pediatrics (STEP) database of practical use.

Among the largely employed substances in medicinal formulations, a particularly careful evaluation is needed in the choice of sweeteners, flavors, plasticizers, solvents, preservatives, and colorants. In neonates, substances such as benzyl alcohol and polysorbate 80 have been associated with increased mortality, parabens have been correlated to hyperbilirubinemia, while aspartame and acesulfame potassium are found to decrease sensibility to insulin [18,19]. Both ethanol and propylene glycol can alter the central nervous system development and be metabolized by the same enzymatic pathway of many common drugs including paracetamol, they may increase the risk of reaching toxic API levels and API accumulation [20]. Recently, it has been reported that the toxicity effects in neonates exposed to lopinavir/ritonavir oral solution (Kaletra^®^), an antiviral drug combination for the treatment of HIV infection, were due mainly to excipient-excipient interaction [21]. In fact, this solution contains relevant amounts of propylene glycol (152.7 mg/mL) and ethanol (356.3 mg/mL), which are both almost exclusively eliminated by metabolic clearance through alcohol dehydrogenase (ADH) [21]. More recently, it was speculated that the amount of ethanol present in the buprenorphine formulation (0.075 mg/mL, containing 30% ethanol, 0.016 mg/kg/day) could in part explain the differences in neonatal abstinence syndrome (NAS) symptoms observed in a clinical trial [22]. In this regard, the U.S. Food and Drug Administration (FDA) recommends that medicines for children less than 6 years old should be alcohol free. In the European Union (EU), common drugs such as ranitidine, furosemide, mannitol, phenobarbital, cotrimoxazole, and paracetamol contain ethanol in their formulations. The revision of the annex to the EC guideline on “Excipients in the labeling and package leaflet of medicinal products for human use” recommends that ethanol should not be included in medicinal products unless its benefits far outweigh the risks associated with alcohol intake [23]. In this context, the EMA proposed a limit of 1 mg/dL ethanol for a single dose of medication and a daily limit of ingestion of 6 mg/kg/day for children under 6 years.

Observations concerning the toxicity of excipients such as polysorbate, propylene glycol, benzyl alcohol, and benzalkonium chloride were already reported in the 1980s. Thrombocytopenia, renal dysfunction, hepatomegaly, and ascites were observed after the administration of a vitamin E supplement containing 9% polysorbate 80 and 1% polysorbate 20 to neonates [24]. Furthermore, preterm neonates administered with parenteral nutrition formulations containing propylene glycol up to 3000 mg/day, underwent relevant side effects such as seizures or intracranial hemorrhage and other effects related to the chemical nature of the substance (hyperosmolarity, lactic acidosis, creatinine), due to their immature hepatic and renal clearance [25]. While products containing high levels of propylene glycol should be avoided under the age of 4 years, as its accumulation may occur due to a higher half-life (e.g., 16.9 h in neonates vs. 5 h in adults), this solvent is worryingly present in intravenous preparations containing dexamethasone, diazepam, digoxin, lorazepam, nitroglycerin, or phenobarbital, thus exposing neonates to harmful concentrations [19]. As reported in Table 1, the acceptable daily intake (ADI) for this solvent is equal to 1 mg/kg and 50 mg/kg for neonates up to 28 days and from 29 days up to 4 years, respectively.

Similarly, in the early 1980s, following exposure to the commonly used preservative benzyl alcohol (130 mg/kg/day), preterm neonates displayed gasping syndrome with metabolic acidosis, bradycardia, and seizures. Since benzyl alcohol partially undergoes hepatic oxidative metabolism to benzoic acid, a reduced metabolic capacity to inactivate benzoic acid to hippuric acid has been suggested as the underlying mechanism of benzyl alcohol toxicity in newborns [26]. In this scenario, benzyl alcohol should be avoided in pre-term or full-term neonates unless strictly necessary because of the risk of severe toxicity, and it may be used for children older than 4 weeks with caution. Nevertheless, the inclusion of benzyl alcohol in the group ADI of 0–5 mg/kg for benzoic acid and benzoates, its thresholds needed to be revised. Indeed, children below 3 years old may not be capable of eliminating benzyl alcohol as efficiently as adults, so the upper limit of the ADI should be considered with caution.

Another important aspect regards the use of natural or artificial sweeteners with the aim to improve palatability and compliance of children. Their use in formulations for neonates is not recommended due to a lack of established safety data [19]. Intolerant subjects may exhibit gastrointestinal symptoms when taking a medicinal product containing lactose. Similarly, sorbitol can also lead to disorders such as diarrhea. Chronic treatments with medicines reached in sucrose may favor the onset of tooth caries. Furthermore, the use of natural sweeteners should be avoided in patients affected by type 1 diabetes mellitus. Concerning artificial sweeteners, aspartame, as a source of phenylalanine, may be harmful in subjects affected by phenylketonuria, while saccharin-containing drugs should not be administered to children with a known sulphonamides allergy. Finally, it was found that saccharin can increase the risk of developing bladder cancer [19].

Worryingly, although the awareness of the several potential side effects associated with the use of the abovementioned substances in children, their exposure to them is still remarkable, as confirmed by a European observational study. The presence of potentially harmful excipients such as parabens, benzoates, benzalkonium chloride, saccharin, sorbitol, propylene glycol, ethanol, and polysorbate 80 was found in 31% of prescriptions in neonatal intensive care units (NICUs) from 21 countries. Parabens were the most frequently administered, followed by propylene glycol and benzoates [27]. More recently, to perform a risk/benefit assessment of the administered medications, a retrospective study on children hospitalized at Rigshospitalet, (Copenhagen, Denmark) was performed by Valeur et al. [28]. This project, namely Safe Excipient Exposure in Neonates and Small Children (SEEN), and other studies, such as the European Study on Neonatal Exposure to Excipients (ESNEE), aimed at generating information for integrating already existing repositories such as the Safety and Toxicity of Excipients for Pediatrics (STEP) database. The STEP database [29] was created by the European and United States Pediatric Formulation Initiatives (PFIs) in 2014, to address the shared need of the scientific community to effortlessly access the available safety and toxicity information of excipients. Table 1 provides an overview of tolerance limits as currently proposed by the EuPFI database [30].

**Figure 3:**
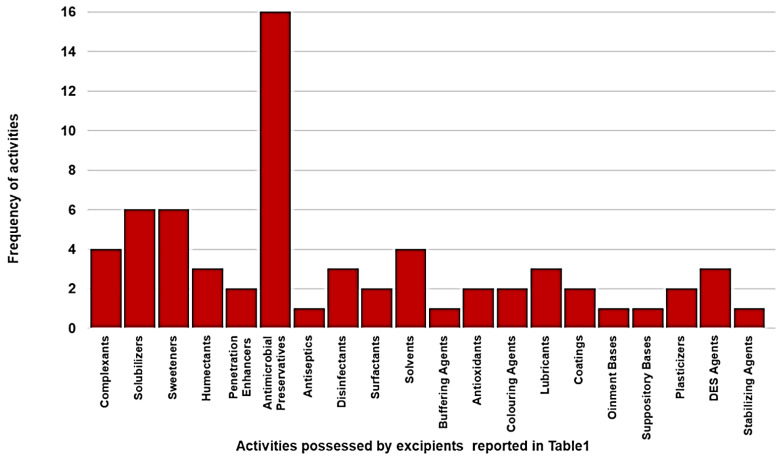
Main activities of excipients reported in Table 1 and their frequency (DES = dispersing, emulsifier, suspending).

Interestingly, Figure 3 shows how many excipients, among those reported in Table 1, display a specific effect.

Particularly, Figure 3 evidenced that most parts of the available and employed excipients are antimicrobial preservatives, thus underling that maintaining the integrity of drug formulations is one of the most important challenges of formulators. The CHMP guideline in excipients in the dossier for application for marketing authorization of a medicinal product asserts that “*excipients to be used in formulations for the paediatric population should be selected with special care and possible sensitivities of the different age groups should be taken into consideration*” [30]. Despite this, since 2010, while new medications authorized in Europe must specify quantitative details of excipients in the Summary of Product Characteristics (SmPCs), many pediatric formulations licensed before the European Pediatric Regulation contain excipients not recommended for use in neonates [31]. The continuous evolution of the regulatory aspects in amending dated thresholds for the amounts of excipients to be used shows all the limitations of these attempts to convert the awareness of the existence of several problems into guidelines and clinical practices, since values as such do not warrant safety.

In summary, the formulator should first consider whether any excipient is required when developing a formulation for children and mostly for neonates. For example, to avoid the use of antimicrobial excipients, even an oral liquid medicine might be designed as a sterilized unit dose. Notably, preservative-free formulations should be considered whenever possible. Another class of excipients whose use should be carefully evaluated are colorants. They are generally not needed for this population and if intended for administration via the non-oral route—along with sweetening and taste-masking agents—may not be necessary. Overall, the number of excipients and their concentration should be the minimum requirement to realize a product with good performance, stability, and dose uniformity, without forgetting children’s compliance.

### Focus on Flavors for Paediatrics: How to Select the Right One

Within the Pediatric Regulation, according to the PIP guidelines, during the design of a new pediatric product, the study of the organoleptic properties is a mandatory step. In fact, when defining the characteristics of a product for children, masking any unpleasant tastes of the API is of great relevance, since good palatability will prevent the pragmatic approach to obscure the unpleasant taste of medicine by mixing it with food. Employed for decades, the most common taste modification method consists of adding sweeteners and flavors, through the “trial and error” approach, to test which combination fits well. On the other hand, a too pleasant taste may increase the risk of misuse in children, so a “neutral” taste is considered the most appropriate choice.

In this context, the formulator must consider that flavors are often a complex mixture of chemical substances, whose composition is not always known. Not knowing exactly the chemical composition of a flavor, adds further complications regarding the compatibility of the flavor with other excipients, as well as concerning its possible toxicity and its level of tolerability (sensitization and risk of allergies). Additional safety concerns may also arise for liquid flavors containing ethanol or propylene glycol. A risk-based approach should be used for the selection of flavors, in the case of pediatric formulations, and it would be desirable to provide a list of 1st line, 2nd line, etc., flavors choices. The right choice should consider both the age of the target children and all involved regulatory aspects. The regulations in the flavorings sector are quite heterogeneous; regardless, we have provided a useful list for suppliers to evaluate if all the conformities have been compiled for a safe selection of aromas. The herein reported Table 2 could represent a checklist that could help in the choice of a flavor usable in a pediatric formulation.

## 4. Appropriate Medications for Pediatrics: The Paradigm Shift towards Oral Solid Dosage Forms

The choice of a certain dosage form has also a significant impact on the excipient options. Unfortunately, many of the excipients that can potentially induce health problems in pediatrics are often strategic to produce liquid products, to increase drug solubility in them, their palatability, and for providing stability. Consequently, within the scientific community, there is an ongoing effort to develop feasible formulations for pediatrics.

The ideal formulation should permit the administration of a large range of doses and should be acceptable for children of different ages. Certainly, traditional oral medicinal products for pediatrics are liquid dosage forms (i.e., solutions, drops, and suspensions), as they are easily administered, and the dosage is adjustable based on the patient’s weight. However, regarding the suspensions, the risk of under- or –overdosing following an inadequate shaking is a well-known issue. Furthermore, suspensions are difficultly feasible for their physicochemical characteristics, along with their tendency to foaming, sedimentation, and sticking of the suspended drug to the primary container or to the measuring device. Concerning oral drops, even if they could allow administering medicinal products in low doses and small volumes, since the risk of counting an incorrect number of drops is high, their feasibility for pediatrics should be considered only for API with a wide therapeutic window. In syrups, physical, chemical, and microbial fluctuations are difficult to overcome, and the control of drug release is hardly achieved. In any case, it is worth considering the key factor of palatability, which is particularly challenging especially when small volume amounts are necessary. Furthermore, dosing errors due to the low accuracy in administering them through spoons, cups, or syringes have been confirmed by several studies [37,38].

**Figure 4:**
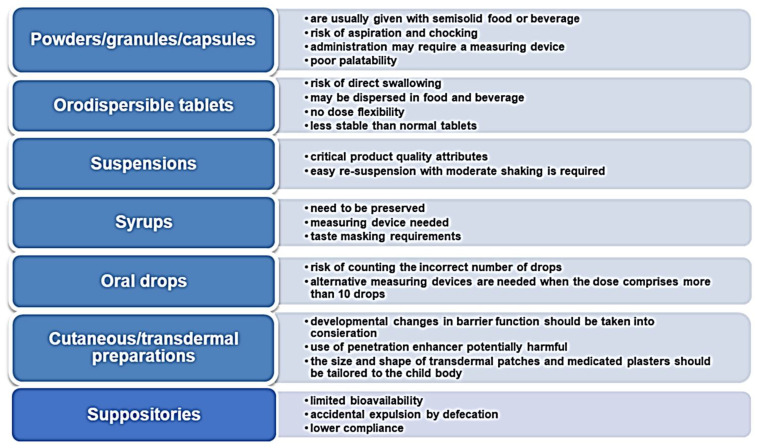
The main usability issues and concerns of common pediatric dosage forms.

Hence, despite liquids historically representing first-choice formulations, in recent years there has been a revival of oro-dispersible tablets, multi-particulates, and powders due to the awareness that oral solid dosage forms may circumvent stability issues, bulkiness in formulation, and exert control of drug delivery. Oro-dispersible tablets were the first solid formulation successfully used in children. They partially overcome the insurmountable obstacle of swallowing, but, although this facilitation, they still show the limitation of no dose flexibility as in the case of conventional tablets, worsen by their fragility, which hampers their splitting. Their low hardness also requires special packaging to avoid breakings during handling and transport [39]. Moreover, children may not be able or willing to take the tablets as intended, or time residence variability may lead to fluctuations in drug bioavailability. Oral powders and granules provide greater dosing flexibility than single-unit oral dosage forms and are easy to swallow. Unfortunately, since they are often judged as being poorly palatable, they are usually added to food or drink by caregivers, even when such practice is not recommended in the leaflet. Pellets, being a flexible multi-unit dosage form, could also be of interest in the development of pediatric formulations, however, they require solvent before extrusion and spheronization and are more suitable when controlled release of the drug is desired [40]. The main issues and concerns regarding the use of the most common pediatric solid and liquid dosage forms are summarized in Figure 4.

In this regard, the European Paediatric Translational Research Infrastructure (EPTRI) project commissioned a case study aimed at collecting information about children and adolescents’ experiences and preferences for oral dosage forms across various European countries (the United Kingdom, Italy, Spain, Albania, Romania, the Netherlands, the Dutch-speaking part of Belgium, Czech Republic, and Sweden) [41]. Overall, liquids were the most used and favored dosage forms. They were widely selected by children less than 12 years old and by those without any chronic condition. On the contrary, tablets and capsules, were mostly chosen by adolescents and children taking medicines more frequently. Granules were the last option among adolescents, thus resulting in the least appreciated formulation together with the oro-dispersible films, probably due to the lack of acknowledgment of this quite new dosage form. Intriguingly, children, aged 6–12 years, whose selection was based on their perception instead of experience, chose effervescent tablets, MTs, and oro-dispersible tablets as their first three choices, confirming a paradigm shift from the belief that all young patients prefer liquid dosage forms. Surprisingly, among Dutch people, a positive attitude towards MT emerged [42]. Overall, these findings seem to support formulators to develop more pediatric medicines in these types of dosage forms.

Lately, the study of oro-dispersible films for pediatric use is also gaining interest among scientists. However, they are not explicitly described in the EMA guideline and several limitations persist, including the difficulty in masking unpleasant tastes, the impossibility of performing controlled release, and poor drug loading. Additionally, their restricted suitability to topic release rather than systemic are issues far from being solved. Moreover, high production cost together with the lack of harmonized test methods, are limiting their use [42].

In the past, the main concern preventing the use of solid dosage forms in young children was the fear of choking. However, several studies focusing on MTs demonstrated the ability of infants to take these dosage forms safely. Notably, Klingmann et al. clearly demonstrated that the administration of multiple MTs was feasible, well-tolerated, and safer even than that of syrup, for all children starting from 6 months, finally shifting the paradigm from liquid to solid dosage forms [43,44,45]. For example, 4 mm MTs were successfully administered from 1 year of age, while 2 mm MTs from 6 months of age, and rapidly dissolving 2 mm MTs in the pre-term age. Although the repeatability of these findings at home remains to be proven in a larger scale of pediatrics, a study in a domiciliary setting by van Riet-Nales et al. showed that 4 mm MTs were preferred the most over oral powder, suspension, and solution [46].

### 4.1. Mini-Tablets

In recent years, the regulatory framework supported the design of novel technologies for the manufacturing of age-appropriate formulations. These studies have resulted in significant advancements in the development of new therapeutical approaches, including dispersible tablets, oral films, and MTs, and in the arrival on the market of new dosing devices (e.g., medicated straw and MT dispensers). MTs are not strictly defined by regulatory guidelines. They are considered only as smaller typical tablets with diameters ≤ 3 mm comprising conventional MTs (coated or uncoated), and oro-dispersible MTs. They possess a wide range of applications since they can perform, as conventional tablets, both drug immediate release and modified drug delivery, including prolonged release, delayed release, pulsatile release, bimodal release, and targeted release [47]. In addition, MTs represent a very flexible drug delivery tool since they can be administered as a single unit or as an ensemble of multiple units. Since counting the right number of small units could be difficult for patients or caregivers, new dosing devices have been patented and already launched to the market to measure a defined number of MTs without making errors.

Regarding MT biopharmaceutical features, it has been proven that MTs can achieve the same independence to gastrointestinal transit as that of traditional multi-particulate dosage forms [48]. These systems show considerable advantages, derived in part from being a multi-unit dosage form and in part from their peculiar manufacturing process. All the main advantages and disadvantages of MTs are summarized in Table 3.

Compared to other multi-unit dosage forms, such as granules and pellets, MTs are more reproducible and uniform. MTs possess a well-designed size, shape, smooth surface, low degree of porosity, and high mechanical strength. Indeed, the reduction in tablet size leads to higher mechanical strength with a low capping tendency. On the other hand, drug-loading capacity depends on MT’s weight that could be only near 6 mg, thus strongly limiting the drug dose per tablet. The desired dosage regimen can be easily provided by changing the ratio between the immediate-release units and the prolonged-release units. Many examples of modified release MTs are reported in the literature, but here we focused on the latest advancements on formulations mainly intended for pediatrics and on their manufacturing techniques.

Lately, pharmaceutical companies have invested in MT marketing and several pediatric products are already present on the market. A few examples of commercially available MTs are reported in Table 4. Lamisil^®^ oral granules (Novartis) and Orfiril Long^®^ (Desitin) contain 2 mm MTs and are dispensed in stick packs and capsules. They are intended to be administered to children by sprinkling on food. Pancrease MT^®^ contains MTs enclosed inside a capsule.

#### 4.1.1. Mini-Tablet Manufacturing Techniques: Compression Methods

**Figure 5:**
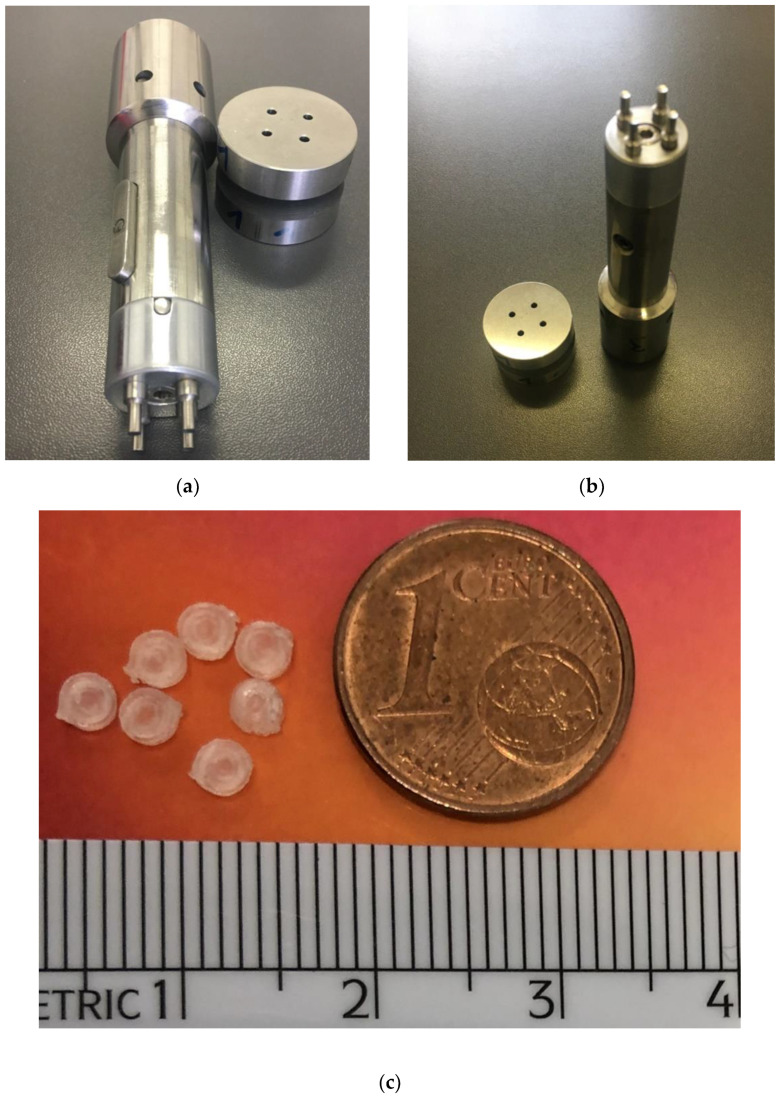
Multiple-tip punch with the correspondent die for mini-tablet manufacturing (**a**,**b**); typical dimension of mini-tablets (**c**).

As in the case of the conventional tablet manufacturing process, MTs could be prepared by direct compression or dry and wet granulation of the powder blend. The method of direct compression to craft MTs is particularly appealing for its convenience and feasibility, being the shortest one. However, conditions such as low or high doses of API present a challenge in this respect. Most APIs show poor compressibility, which reduces the hardness of tablets in the case of a large proportion of API. On the contrary, low fractions of API are difficultly blended with homogeneity. In these cases, granulation represents the best choice, and it can be performed by wet granulation, or by roller compaction (dry granulation method) when the API is thermo-labile and moisture sensitive. Alternatively, granules can be obtained from the melt extrusion method. MTs can be produced by eccentric or rotary tablet press machines equipped with multiple punches. Notably, MTs require more attention regarding production parameters such as flow, particle size distribution, and tooling dimensions since the lower size is an additional critical factor for the success of the compression process. Flemming and Mielk recommended that to avoid insufficient filling during MT production, the maximum particle size should not exceed 1/3 of the die diameter [49]. This principle was confirmed in a study carried out using lubricated microcrystalline cellulose (MCC) powder blends and dry-granulated blends pressed with multi-tip tooling of 1.7 mm tip diameter [50]. Moreover, it is also worth considering the hole length and diameter. The diameters of dies range from 4 mm to 2 mm. In the former, the bulk flow rate increases with the hole length, whereas in the latter, it decreases with the elongation of the hole. The narrow volume of the dies requires an excellent powder flowability, therefore, ambient humidity and the narrowest particle size range are parameters to be checked to prevent occlusion. Once the die is uniformly filled, the compression is exerted by punches. Multiple-tip punches are available as mono-blocks or as multi-piece assemblies. The former is assembled with a separated cap, fixed to the punch body through external or internal staples, and the other consists of a mono-block easier to clean, but that needs to be entirely replaced when the edges are damaged by erosion (Figure 5).

Since the amount of powder in tight contact with the wall of die and punches is greater, MTs show a smooth surface and a homogenous density with a poor danger of capping even at high drug cargoes. On the other hand, due to the small diameter of the tips, they can easily deform and break if an excessive compression force is applied. Moreover, due to the higher surface area/weight ratio, MTs undergo higher ejection forces, which may cause sticking. MTs can be further coated by pan coaters or fluid beds. Typically, MTs are fluidized in conventional Würster fluid beds, while perforated pans should be modified, to avoid MTs falling through the perforations [51]. However, since pediatric formulations should preferably be composed of the fewest possible number of ingredients to avoid safety concerns, as the EMA guideline suggests, the coatings should be considered only when relevant. The risk that a pediatric patient may inadvertently chew a modified-release preparation and the resulting harm is worthy of serious consideration.

Therefore, fast-dissolving MTs are the best appropriate dosage form for pediatrics. Among excipients suitable for manufacturing fast-dissolving tablets, mannitol represents the first-choice material, due to its low hygroscopicity, good disintegration properties, and sweet taste. However, to perform oro-dispersible MTs by direct compression, the technological properties of this excipient need to be improved, and the employment of co-processed mannitol excipients may be required.

##### Case Studies on Oro-Dispersible MTs Obtained by Compression Methods

The first published study aimed at preparing oro-dispersible MTs was performed by Stoltenberg et al. in 2011 [52]. They investigated the suitability of five co-processed mannitol excipients to craft MTs containing hydrochlorthiazide, a diuretic drug used in pediatrics. Notably, although hydrochlorthiazide is approved for pediatric patients, and it is included in the WHO’s Model List of Essential Medicines for Children, the lowest dose currently on the market (12.5 mg) is not suitable for infants and toddlers, that require from 1 to 5 mg. In this context, the study of Stoltenberg and co-authors represents an attempt to solve the longstanding problem of crushing tablets, by optimizing the preparation of oro-dispersible MTs. Among the ready-to-use tableting excipients, Ludiflash^®^, Parteck^®^ ODT, Ludiflash^®^, Pharmaburst^®^ 500, Prosolv^®^ ODT, and Pearlitol^®^ Flash were considered. Concerning the compactability, at compression forces ranging from 5.5 to 8.5 kN, Parteck^®^ ODT was revealed to be the best one, while Pearlitol^®^ Flash was the less appropriate, probably due to the presence of maize starch. Unfortunately, while Parteck^®^ ODT showed the longest wetting time (WT), Pearlitol^®^ Flash and Ludiflash^®^ displayed the shortest (<3 s for a 5.5 kN compression force for the latter). All the 1 mg hydrochlorthiazide MTs complied with the requirements of mass variation (MV) and of drug content uniformity (DCU), but the acceptance values (AVs), despite being under the Ph. Eur. limit (<15), were quite high. An explanation may rely on the drug segregation induced by the size difference between the hydrochlorthiazide powder and the excipients. They concluded that Ludiflash^®^, which in addition to mannitol contains crospovidone as a disintegrant, and polyvinyl acetate as pore former, provided the best co-processed mixture. More recently, the same authors investigated the manufacturability of MTs using galenIQ™721, an agglomerated isomalt containing the two disaccharides, 6–*O*-α-D-glucopyranosyl sorbitol and 1-*O*-α-D-glucopyranosyl D-mannitol dihydrate in a ratio of 3:1 [53]. Like Ludiflash^®^, galenIQ™721 is a spherical shape powder with a mean D_50_ close to 130 µm, showing good flowability. All hydrochlorthiazide formulations, mixed with 0.5% silicon dioxide (SiO_2_), fulfilled the Ph. Eur. requirements for the disintegration test (180 s), while those containing the super-disintegrant Kollidon^®^ CL-SF satisfied the FDA criteria (30 s). A further increment of flow aid up to 2% was detrimental for the disintegrant rate for isomalt without disintegrant, this can be related to the capability of SiO_2_ to swell and form hydrogels at certain concentrations. Therefore, the presence of the super-disintegrant (Kollidon^®^ CL-SF) was necessary to accelerate the disintegration process, since 2% SIO_2_ was paramount to reach an AV ≤ 15. Regarding dissolutions studies, the drug release was faster with isomalt than Ludiflash^®^, as it occurred through swelling to a smaller extent. In addition to hydrochlorthiazide MTs, the authors also prepared 1 mg enalapril maleate MTs. Enalapril is an angiotensin-converting enzyme (ACE) inhibitor commonly administered off-label in patients <20 kg. In contrast to hydrochlorthiazide, the formulations with isomalt showed an AV ≤ 15 only without SiO_2_. Probably, the presence of the flow aid supported the segregation of the API, thus affecting the drug content uniformity. Moreover, in contrast to hydrochlorthiazide, the enalapril formulation with Ludiflash^®^ did not achieve the required content uniformity. Regardless, since enalapril has gained importance in recent years among the scientific community, further efforts have been made to develop enalapril-containing oro-dispersible MTs for pediatric use [54]. In addition to mannitol and isomalt, lactose co-processed excipients were also employed to design MTs by direct compression. Tablettose^®^ 80, Microce- Lac^®^ 100, and StarLac^®^ mixtures were examined. Formulations with 0.1 mg of enalapril maleate including StarLac^®^ (lactose and starch) presented good quality parameters complying with the official parameters of hardness (39 N), friability (<1%), disintegration time (28 s), drug content uniformity (103.6%), and wetting time (23 s). Therefore, this formulation could be considered eligible for being manufactured on an industrial scale.

If the active ingredient is extremely potent, the problem of content uniformity becomes essential and of great concern. Regarding this, risperidone is a potent antipsychotic agent, whose starting dose is 0.25 mg/day in children weighing less than 20 kg, endowed with bad compaction properties and poor flowability. To address these issues, El-Say et al. optimized the excipient combination by performing the experimental Box–Behnken design [55]. Oro-dispersible MTs were prepared by direct compression, after the selection of appropriate excipients with low toxicity for children. The most suitable powder blend combination consisted of 0.5 mg risperidone, 2.3 mg mannitol, 1.7 mg MCC (Avicel^®^), 0.5 mg croscarmellose sodium (Ac-di-Sol^®^) as super-disintegrant, a mixture of 50 μg SiO_2_ (Aerosil^®^ 200: Aerosil^®^ 300, 1:1, *w/w*) as a glidant, 25 μg aspartame, and 25 μg peppermint oil. This formula satisfied the requirements for friability, uniformity of mass, and drug content. Disintegration time was longer in a skimmed milk model than in simulated saliva, so the requirements for oro-dispersible MTs were fulfilled only in the second condition.

As proof of the empiricism that regulates the use of the pediatric dosage forms, the commercially available 10 mg hydrocortisone tablets are quartered scored, to allow by their splitting the administration of the 2.5 mg dose to children as replacement therapy in primary, secondary, or acute adrenal insufficiency. Concerning this, Madathilethu et al. proved that, although this practice has been in place for years, 41% of the quartered tablets failed to meet the specifications of weight uniformity and consequently the coefficients of variation from the mean drug content ranged from 7.3 to 19.3% [56].

To overcome this unacceptable variability in hydrocortisone content, the authors manufactured 3 mm MTs using the wet granulation method to improve the flow properties, thus ensuring the uniformity of the die fill. Recently, DIURNAL LTD applied for a PUMA to EMA, concerning a hydrocortisone formulation consisting of granules in capsules for opening, intended for all pediatric populations [57].

For APIs which require high dosage, the small size of an MT, whose weight ranges from 5 to 20 mg, may be insufficient to contain all drug content in one unit. This is the case of loratadine, an antihistamine agent used to reduce the symptoms of allergy. For children over 6 years, the recommended daily intake of loratadine is 10 mg given through a tablet, for those under 6 years a syrup is used. In this case, being the API amount quite considerable, a single dose must be portioned in more units, as performed in the study of Gulnur Yeleken et al. where a dose of 5 mg loratadine was subdivided into 10 units of MTs [58]. In this study, to investigate the feasibility of MTs by direct compression, several formulae were considered. The most satisfying blend contained MCC as a diluent, as well as corn starch and croscarmellose sodium as disintegrants.

Although the attempts made in recent years evidence growing interest from the scientific community in bespoke pediatric formulations, only lately the efforts in formulation design have paid due attention to the toxicity information collected by the regulatory agencies. An example in this context is represented by the study of Freerks et al. [59]. The authors finetuned furosemide MTs with a mass of 2.5 mg, obtained by a rotary press equipped with 4 mm round punches. Particularly, uncoated oro-dispersible MTs were prepared with great attention given to the selection of the excipients and considering only those included in the STEP database. Among the tested excipients, milk powder showed a good binding capacity but a sharp increase in the disintegration time. D-mannitol and Ludiflash^®^, which contain 90% of mannitol, were tested as filler to reach fast disintegration but were subsequently discarded considering the laxative effects that they could exert. On the contrary, lactose and Ludipress^®^ were regarded as safer, being lactose present also in milk, though their cariogenic potential and their unsuitability for lactose intolerant and galactosaemic patients. Moreover, Emdex^®^, which is a mixture of 95% glucose and dextrates, was regarded as a safe excipient as a disintegrant, crospovidone (Kollidon^®^) appeared to be the best one, while, as sweetener, the not cariogenic sucralose, was preferred. To minimize risks, only natural flavors were employed. Interestingly, the authors performed an additional test to investigate the friability in a simulated multi-dose container. Additionally, a customized disintegration test in a small amount of fluid (equal to a spoon) was performed to mimic a possible situation before administration, while dissolution experiments were carried out simulating the gastric and small intestine conditions of 1-year and 5-year-old children. The dose was calculated using WHO Child growth standards and the dosage form was administered alone or together with apple juice, milk, or yogurt. Collectively, in this study, not only great attention to the toxicological aspects in the selection of ingredients was paid, but also the technological controls were performed considering the MT characteristics and the possible way of administrations, regardless of the instructions presented in the European Pharmacopoeia. Overall, this study could be used as a suitable formulation platform for PUMA applications.

With the aim of improving the dissolution performances of poorly soluble APIs, the spherical cocrystallization technique was performed to develop MTs loading piroxicam [60]. This approach leads to an increase in flowability by forming spherical aggregates and, at the same time, to the enhancement of the apparent solubility of API, due to its cocrystallization with a water-soluble molecule. To this end, piroxicam-ferulic acid cocrystals were formed in an acetonitrile solution containing the two molecules in a 1:1 ratio. After crystals formation, their agglomeration was promoted under agitation in a bridging liquid (chloroform: ethyl acetate) and in an ice bath to attain a good yield of agglomerates. The cocrystals showed improved tabletability associated with higher plasticity and a fair flowability due to size increase.

To date, only one study has regarded preclinical investigations [61]. The anticancer agent lapatinib, a tyrosine kinase inhibitor, has been approved for the treatment of human epidermal growth factor receptor 2 (HER2)-positive breast cancer. HER2 is overexpressed in many childhood cancers, such as medulloblastoma, making this drug particularly attractive also for pediatrics [62]. Unfortunately, this small molecule is poorly soluble in water; therefore, the formulation design must consider both the preparation of a flexible dosage form and the identification of the best method to increase the lapatinib solubility and thus its bioavailability. To this end, amorphous solid dispersions of lapatinib were firstly prepared by spray-drying an organic solution containing hydroxypropyl methylcellulose E3 (HPMCE3) or hydroxypropyl methylcellulose phthalate (HPMCP) in a 1/3 drug/polymer ratio. Subsequently, to MT manufacturing by direct compression, 20% of the spray-dried powder was combined with croscarmellose sodium, as a disintegrant, and with MCC (Avicel^®^ PH 200). Solid-state analyses highlighted the presence of acid-base interactions between lapatinib and HPMCP which probably were responsible for the highest stability of this amorphous dispersion compared to that containing HPMCE3, which after 30 days evidenced drug crystallization. The 2 mm MTs with HPMCP showed a mean drug content of 107% and a quite high AV. A pharmacokinetic study in juvenile pigs highlighted a ten-fold higher AUC for lapatinib-HPMCP MTs versus MTs containing lapatinib in the crystalline form.

#### 4.1.2. Hot Melt Extrusion Technique

Hot melt extrusion (HME) is a process for manufacturing amorphous solid APIs dispersions, also employed to produce taste-masked MTs. HME allows the enhancement of the dissolution rate of poorly water-soluble APIs and depending on the polymer used, it can provide delivery systems with either modified or immediate release. In this regard, 5 mm diameter mini matrices made of Eudragit^®^ E PO (EPO) (a terpolymer based on N,N-dimethylaminoethyl methacrylate with methylmethacrylate and butylmethacrylate), and containing molecularly dispersed ketoprofen, were obtained by melting powders at 120 °C [63]. Since ketoprofen melts at 97 °C, the resulting extrudate strands appeared clear and smooth prior to entering the cutting pelletizer, indicating the melt solubilization of the drug in the polymer matrix during heating occurred. It was evidenced that for drug loadings over 50% (*w/w*), excessively elastic and deformable strands were obtained, thus establishing that 40% was the optimum drug content. The resulting MTs passed both uniformity of content and friability requirements and showed a complete drug release within 20 min in the gastric medium. The supersaturated solutions were stable during 2 h of permanence in the dissolution medium as a result of the salt formation between the carboxylic group of ketoprofen and the ammonium group of EPO.

#### 4.1.3. Electrospinning Technique

Electrospinning is a technique that produces nanofibers with a customized diameter by an electrostatically driven jet of either a polymer solution or a molten polymer. Since this approach leads to the formation of porous structures with high surface area, it can be tunable for realizing MTs embedded with poorly soluble APIs. In this context, electrospun nanofiber mats made of PVP and prednisolone (10% *w/w*) were compressed to obtain 2 mm MTs [64]. The tableting partially induced nanofiber fusion and coalescence at the MT surface, and, intriguingly, the electrospun-derived MTs proved good mechanical properties and low friability, without the need for any binding excipient. An improved solubility was attained, and no recrystallization occurred over time, presumably due to the presence of PVP. Moreover, the API dissolution rate was very fast, due to the large superficial area. The overall merit of this formulation is the paucity of the number of excipients, although additional studies concerning the residual organic solvent in the MTs are required, and no less important are the scalability issues associated with this technique, whose long time of production is a big limiting factor. Indeed, research studies mostly focused on the single-needle electrospinning method, despite attempts to perform high-throughput electrospinning are emerging [65].

#### 4.1.4. 3D Printing Technique

In the 3DP technique, a solid dosage form is realized through the subsequent deposition of stacking layers of materials, allowing the easy fabrication of individualized medicinal products, eventually containing also multiple APIs [66]. Moreover, 3DP has gained a lot of attention in the pharmaceutical industry since the FDA approved a 3D-printed SPRITAM^®^ (levetiracetam 1000 mg) fast-dissolving tablets for the adjunct treatment of myoclonic seizures and primary generalized tonic-clonic seizures. There are various 3D printing methods employed for drug product manufacturing that are used based on the materials, equipment, and solidification. HME, mentioned above, is one of the techniques which is used for 3DP [67]. In the pharmaceutical industry, the main employed 3DP techniques include fused deposition modeling (FDM), selective laser sintering (SLS), stereolithography (SLA), drop-on-solid (Dos), and pressure-assisted microsyringe (PAM). In any case, the process begins with the creation of a digital file using computer-aided design (CAD) software, which defines the geometry of the product. Particularly, while the most widely employed 3DP technique is FDM, PAM displays several advantages. Starting from a gel-based formulation, PAM does not require a melting phase, thus preventing possible undesired degradation reactions of thermolabile APIs. Additionally, the gel can also be easily prepared in hospital pharmacies, thus rendering this approach feasible also in a small manufacturing laboratory.

##### Case Studies on MTs Obtained by 3DP

El Aita et al. performed 10 mm tablets for pediatric use by PAM printing technique [68]. As a model drug, the authors selected levetiracetam, which is an anti-epileptic medicine used to treat seizures in epilepsy. The hydrophilic drug was dissolved in water and a PVA-PEG gel was prepared. The semi-solid formulation was transferred into a printing syringe and tablets with a different number of layers were printed to ensure the correct dose for the respective pediatric sub-population using a 3D Bioplotter. After drying, tablets were characterized for their technological properties. The tablets fulfilled the Ph. Eur. requirements for friability, mass variation, and content uniformity. Due to the high solubility of the polymer matrix, the disintegration time was rapid (close to 90 s), but only the tablets with three layers satisfied the requirement of less than 30 s and could be considered oro-dispersible. A complete release of the API was achieved within 15 min. Although the size of the tablets achievable by 3DP (10 mm) makes them not suitable for neonates, this technique emerged as one of the biggest potentials, and is worthy of mention, since neither mixing of the powders, nor intermediate products are necessary.

Krause et al. investigated the suitability of FDM for developing MTs [69]. Here, HME was used to facilitate the melting, mixing, and extrusion of the API and excipients. Two types of MTs were prepared based on hypromellose (HPMC) or hyprolose (HPC) and loaded with caffeine or propranolol hydrochloride, which were selected as model drugs. The melting temperatures were 170 °C for HPMC and 140 °C for HPC-based MTs, respectively. MTs with 1.5, 2, 3, and 4 mm diameters were obtained. A diameter-dependent release behavior was observed. Particularly, the smallest was the MT size, the fastest was the release. From the 4 mm MTs made with HPMC, the caffeine release was 80% after 190 min, while propanolol was released at 80% after 140 min. Differently, from the 4 mm MTs made with HPC both API showed almost the same release rate (80% after 160 min and 80% after 175 min for caffeine and propranolol, respectively). The main limitation of this approach consists of the need to heat the ingredients to temperatures above 120°, which may be critical, and cause the degradation of thermolabile API. To limit this issue and to reduce the melt temperatures, materials with a low glass transition temperature (Tg), such as polycaprolactone, are employed. Furthermore, the paucity of polymers with melt viscosity suitable for extrusion represents another challenge of this method.

A similar approach was employed to develop baclofen mini-caplets made of PVA and sorbitol (10% *w/w*) as a plasticizer [70]. Currently, pediatric baclofen formulations on the market are unavailable, and usually oral suspensions have been prepared from immediate release adult tablets. Neat PVA resulted in processing difficulty, due to its high melt viscosity due to strong hydrogen bond interactions between the polymer backbones. The presence of sorbitol was mandatory to weaken these interactions and favor an easier extrusion. Extrusion was carried out at high temperatures (close to 190 °C) and, although it was below the baclofen melting point, API melt-solubilization occurred, as demonstrated by the solid-state characterization, which did not result in crystals. In vitro disintegration time was over 20 min, attributable to a surface erosion rather than a media uptake mechanism. Surface erosion also influenced the drug release, which resulted in being governed by non-fickian kinetics. Even if a very high temperature was necessary, the short printing time for a unit (1–2 min) makes this approach appealing for the preparation of customized dosage forms also in a compounding pharmacy.

Personalized MTs by FDM 3D printing technology were developed by Ayyoubi et al. [71]. As a model drug, the authors chose nifedipine, a BCS class II agent, whose dose must be frequently adjusted in both adulthood and childhood patients with either chronic hypertension or hypertensive crises. In their study, the authors prepared both full infill and channeled MTs. The filaments were either purchased on the market and then loaded via passive diffusion, or in-house manufactured and loaded by HME. The MTs printed from commercially available filaments made of PVA and PEG resulted in higher quality compared to those obtained using HME filaments, but they allowed a drug loading of only 4%. On the contrary, even if HME filaments had variable diameters and higher porosity due to a non-homogenous extruded mixture, they permitted a drug loading of 50%. The higher drug loading led to a higher friability and lower hardness of the obtained MTs. The dissolution profiles were influenced more by the composition of MTs than by the surface area. The drug release from MTs containing ethylcellulose was independent of the geometry and incomplete, while MTs containing Kollidon^®^ VA 64 showed a faster release, which resulted in a further improvement in channeled MTs. Collectively, the passive diffusion method proved to possess higher potential than HME to be implemented in the clinical practice within hospitals. The use of a 3D printer equipped with a direct powder extrusion module could represent a valid solution for avoiding the necessity of performing printable filaments through HME. Closer collaborations between industries and hospitals should be implemented to realize the clinical translation of customized 3D-printed MTs.

#### 4.1.5. 3D Printing for Production of Friendly-Shape Medicines

According to the Paediatric Regulation, the evaluation of medicine acceptability is an important aspect that must be considered, to pursue marketing authorization. Therefore, there has been an increasing development of new techniques to produce palatable and pediatric-friendly shape medicines, such as jelly oral dosage forms. Usually, they are prepared with gelatin, glycogelatin, and caseinate, and are adopted preferably for delivering drugs via buccal and sublingual routes [72]. Regarding this, chewable tablets intended for children from 2 to 11 years old were developed through the FDM technique coupled with HME [73]. Interestingly, since their shape was inspired by gummy sweets (HARIBO PLC, UK), they appear as candy-like medicines. Particularly, the filaments were composed of indomethacin, an anti-inflammatory drug, PEG 6000, as a plasticizer, and hypromellose acetate succinate (HPMCAS), which is an enteric coating material dissolving at pH 5.5–6.8 at a 20:20:60 *w*:*w* ratio. The printing temperature (165 °C) was higher than that used to extrude the filaments but necessary to reduce the viscosity of the molten material and allow its flowing inside the nozzle. The 3DP dosage form exhibited a good efficiency in masking the bitter taste of indomethacin. When the chewable tablets were experimented in vivo on 10 adult volunteers, no bitter taste was detected due to the negligible release of indomethacin in the small volume of simulated saliva. Obviously, since children will chew the tablets for a few minutes, thus swallowing them, drug release was also evaluated at pH = 7.4. In this latter case, the drug release was rapid, due to the HPMCAS solubility and not dependent on the tablet shape for tablets of similar weight.

Moreover, in a recent study, an emerging technology, embedded 3D printing, has been used to manufacture chewable Lego™-like bricks [74]. The technique involved the deposition of an embedded phase containing the drug into a liquid embedding phase (reservoir), made of gelatin. Notably, ibuprofen and or paracetamol were suspended in a solution of water, glycerol, gelatin, and Locust bean gum as a suspending agent, obtaining the embedded phases, which were heated to 75 °C, and then placed into the Lego™-like template, in which warm gelatin was previously poured. In the study, the balance between the suspension drug loading, the viscosity, and the time of its solidification within the embedding phase has been optimized. The possibility to achieve simultaneous dosing of two or more APIs at a flexible concentration could be of great clinical utility. In this regard, the authors showed that it was sufficient to print different percentages of patterns, to obtain different doses. Good linearity, between the percentages of the printed patterns and the actual doses of drugs obtained, was demonstrated. Regardless, since at the beginning the dynamic viscosity is higher, for small size design, the shear-thinning behavior of the embedded phase caused a decrease in the extruded ink amount, thus leading to an inferior drug deposition. The release rates of the two model drugs were similar in simulated intestinal fluid (pH 7.2), regardless of their different water solubility, due to the slow dissolution of locust bean gum, which controlled the diffusion of drug molecules, through the formation of a gel phase.

Generally, the chewable dosage forms require many excipients to obtain a product with good organoleptic properties. Indeed, in designing such formulations, the formulator must consider many factors, including taste, odor, flavor, texture, and visual aesthetics of the product, which can lead to a harmful increase in the number of ingredients. Indeed, the 3DP technique has allowed considerable advancements both in the number of excipients to be used and in the dose flexibility. Regardless, since the drug release process and thus the therapeutic effect is still dependent on the children’s chewing ability, intra- and inter-individual variability are the major concerns regarding the chewable tablets. Moreover, there is a lack of evidence about the safety of chewing products in young children and current guidelines only recommend their use over 6 years. Furthermore, concerns were expressed regarding the possible misuse of these medicinal products, which can be greatly appreciated by children that they can be exchanged for sweets.

### 4.2. Low-Dose Micro-Tablets

Generally, MT diameters are around 3 mm, but some authors have expanded the radial dimension up to 6 mm. On the contrary, with Micro-Tablets (MicroTs), tablets of 1.2–1.5 mm diameter are intended [75]. Since a smaller excipient burden is required and more dose flexibility in the clinic is provided, with MicroTs the advantages provided by MTs results further improved, thus making MicroTs suitable to treat also the particularly sensitive subpopulation of neonates. As already mentioned, one of the most important concerns in manufacturing tablets is content uniformity. The risk of failure to comply with this requirement increases markedly for low-weight dosage forms such as MicroTs and/or when a potent API must be embedded. One strategy to obtain homogeneous batches consists of reducing the API particle size by milling, but it may be counterproductive due to the increase in the cohesion forces and the possible API segregation. Nevertheless, MT production requires a superior powder flow and a higher die-hole diameter/particle size ratio; therefore, the dimensions of the powder should be strictly monitored. Indeed, it was found that the MicroT diameter and the API size can greatly affect the drug content uniformity per unit, as confirmed by the study of Mitra et al. Among the various prepared formulations, the smallest MicroTs (1.2 mm), manufactured by direct compression and loaded with 3% *w/w* of 100 µm D_6,3_ ibuprofen, failed to meet the acceptance criteria for the drug content uniformity [76]. The study evidenced that the factor that most contributed to the high drug content variability was the variance in the die cavity filling. Indeed, the authors concluded that to succeed in controlling the weight in the direct compression method, the ratio die diameter/particle size should be >20–30. Subsequently, the same authors tried to overcome the problem by employing high shear wet granulation process [77]. Particularly, three different size grades of ibuprofen were dry blended with mannitol, MCC, HPMC, croscarmellose sodium, in a high shear granulator after passing in a #20 mesh screen. After wet granulation and fluid bed drying, the granules were milled and prior compression, mixed with a lubricant and a disintegrant. The final particle size of blended powder beds was in the range 93–148 µm, while the ratio die-cavity diameter/blend particle size ranged from 8 to 13. In this case, the only batch that unmet the content uniformity criteria was that of the 1.2 mm MicroTs loaded with the 0.67% of coarse API (D_90_ 180 µm), therefore, a dose five times lower than that reported in the previous study. Generally, at high dosages, API particle size had no effect on content variability, while at the same drug loading, increasing API sizes led to less uniform batches, according to theoretical expectations. However, for 2 mm MicroTs, an opposite trend was observed, and the lowest mean potency (77%) was observed for the smallest API particle size. In this case, the use of micronized ibuprofen to minimize granule growth might have contributed to the API segregation, aerosolization, and to granule heterogeneity. To minimize the loss of API, a strategy could be to spray a solution or a suspension of the micronized drug as granulating fluid. In this way, it is theoretically possible to reduce API loss during drying and, meanwhile, to reach a more uniform distribution of the API into the powder bed. To this end, very recently, a nanosuspension of irbesartan was prepared by wet milling and was added with HPMC, as a stabilizer [78]. Four API dosages per single unit were manufactured: 0.01, 0.05, 0.1, and 0.5% *w/w*, corresponding to 0.16, 0.8, 1.6, and 8 µg. By a nanosuspension-based high shear granulation process, 1.2 mm MicroTs with low drug content variability in all the evaluated dose strengths were achieved. On the contrary, the use of the micronized API, added along with the intra-granular powder blend, failed to meet the content uniformity criteria at the lowest doses. On the contrary, the API nanosuspension provided MicroTs even at the lowest dose strength per unit of 0.16 µg, which means a dose flexibility of 0.16 µg increments.

The use of interactive powder mixtures has been proposed as an alternative approach to obtain high dose accuracy with API concentrations equal to 1% (*w/w*) [79]. Interactive mixtures are typically achieved by mixing free-flowing coarse carrier particles with cohesive micronized API particles for the time necessary to allow sticking to the carrier surface following the establishment of adhesion forces. Two-millimeter MicroTs were prepared after having mixed spray-dried mannitol (around 200 µm) with micronized sodium salicylate (<10 µm) as a model drug, for 48 h in a Turbula mixer. The establishment of the interactive mixture was measured by the determination of the homogeneity of samples withdrawn at different levels into the powder bed for up to 48 h. Usually, the degree of homogeneity increases with increasing mixing times and levels out around 24 h with a relative standard deviation of 3–4%. The MicroTs complied with the uniformity of content requirements with a drug content per unit of 80 µg (1% *w/w*), and the AV values were much lower than 15. An exhaustive list of the recently prepared MTs and MicroTs has been included in Table 5.

**Figure 6:**
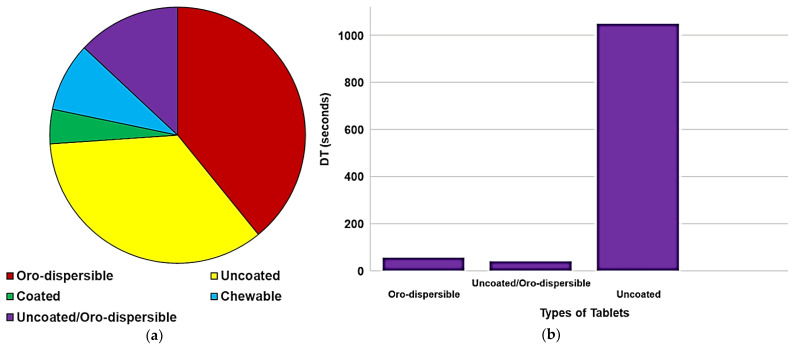
Distribution of the main typologies of the developed mini- and micro-tables formulations (**a**); (**b**) average DT values of oro-dispersible, uncoated/oro-dispersible and uncoated micro- and mini-tablet formulation reported in Table 5.

To provide readers user-friendly tools to easily analyze the data reported in Table 5, some of them are represented graphically and shown in Figure 6.

Particularly, Figure 6a shows the distribution of the main typologies of the developed mini- and micro-tables, including oro-dispersible, uncoated/oro-dispersible, uncoated, coated, and chewable dosage forms, while Figure 6b shows the average DT of oro-dispersible, uncoated/oro-dispersible, and uncoated micro- and mini-tablet formulation reported as a bar graph. Interestingly, Figure 6a shows that the most developed micro- and mini-tablet formulations are the oro-dispersible and uncoated ones, while Figure 6b highlights that the DT dramatically increases in the uncoated tablet not definable as oro-dispersible.

## 5. Clinical Trials

The feasibility of clinical trials involving a pediatric population is particularly challenging. There are very few studies aimed at investigating MTs. One of these regarded a novel prolonged-release melatonin MT formulation (Slenyto^®^, Neurim Pharmaceuticals Steinhausen, CH), which was tested in a randomized, double-blind, multicenter study in children aged 2–17.5 years old (*n* = 125) [80]. The MTs were able to sustain a due drug blood concentration for about 9 h, thus allowing to reduce insomnia in patients affected by autism spectrum disorder. The 3 mm MTs, intended to be swallowed as a whole, were effective in improving the total sleep time, thus also enhancing the quality of life of caregivers.

Recently, an ongoing open-label phase II/III trial in children aged 3–11 years old infected with hepatitis C virus was conducted to assess the pharmacokinetics, efficacy, and safety of ombitasvir, paritaprevir, ritonavir, and to investigate their co-administration with or without dasabuvir and with or without ribavirin [81]. Due to the large variance in weight among the target population requiring a highly variable dosage of API, the drugs under study were formulated in MTs. The median duration of the therapy was 85 days and the high treatment adherence (>90%) confirmed the good acceptability of the children-friendly MT formulations, which were successfully administered in most cases. Unfortunately, although the results underscored the suitability and efficacy of the new regimen, as for most clinical studies in pediatrics, the main limitation of the study is the paucity of the patients enrolled (*n* = 26), as for many clinical studies in pediatrics.

Since randomized controlled studies are often not feasible when a small population of individuals is examined, as recommended by the ICH E11 (R1) pediatric guideline, alternative study design options are suggested, including pediatric extrapolation, single-arm studies, the use of digital registries for indirect comparisons, withdrawal designs, and quantitative or Bayesian approaches [11].

Additionally, to overcome the difficulties in recruiting patients for clinical research studies, the European Commission has funded three international multidisciplinary research consortia under the FP7 Health projects framework to identify promising approaches. ASTERIX (Advances in Small Trials Design for Regulatory Innovation and Excellence), IDEAL (Integrated Design and Analysis of Small Population Group Trials), and InSPiRe (Innovative Methodology for Small Population Research) are the acronyms identifying such consortia. It is certain that these initiatives, like other ongoing collaborative pediatric networks, such as the European Network of Paediatric Research at the EMA or the Innovative Medicines Initiative Conect4children project, will incentivize the development of more pediatric studies.

## 6. Manipulation of Pediatric Medicinal Products: The G. Gaslini Children’s Hospital Experience

Pharmaceutical industries do not consider pediatric medicinal products attractive, so the design and production of formulations for children take a back seat to that of dosage forms for adults, which are of primary importance. Consequently, the manipulation of the dosage form created for adults may be necessarily undertaken at the point of administration to provide the prescribed dose. Worryingly, even if justified by the absence of suitable commercial formulations, this alteration of the original dosage form leads to the common and questionable practice of administering extemporaneous or magistral preparations and off-label medications. For more clarity, with an unlicensed drug, a medicinal product is prescribed for human use, with no granted marketing authorization by the countries’ licensing authority. Differently, a licensed drug used outside its authorized age group of use, indication, dosage, route of administration, or frequency is referred to as an off-label prescription. The preparative procedures and the calculations made to prepare the pediatric doses could result in an inaccurate dosage, which together with the inherent unknown effects on the stability and bioavailability of the modified drug formulation represent a source of potential therapeutic error. The EMA reflection paper states that “*manipulation of adult medicinal products for paediatric use should be the last resort*”, but at the same time underlines that “*this is recognized as an unavoidable and necessary operation in many cases*” [3]. Numerous off-patent compounds can be found on the World Health Organization (WHO) Model List of Essential Medicines List for Children (EMLc), which consists of a list of the most efficacious, safe, cost-effective medicines for priority needs in children (0–12 years) [82]. In a study by delMoral-Sanchez et al., a quantitative evaluation of the age appropriateness of the commercial oral formulations listed in EMLc 7th Edition (2019) was performed [83]. The study highlighted that the percentage of oral dosage forms that resulted as authorized age-appropriate medicines by the EMA is only 52%. Many of the APIs in EMLc are also included in the Inventory of the Needs for Paediatric Medicines filed by the EMA [84]. Since many of them are off-patent, they could represent a great opportunity for obtaining PUMAs, if only there is more interest from pharmaceutical companies. In an observational study, based on 117,665 oral administrations over 1 year in a French pediatric hospital, it was concluded that young children are still commonly treated with unlicensed drugs [85]. Here, we reported the experience of G. Gaslini Children’s Hospital (Genoa, Italy) by listing the most frequently orally administered drugs manipulated in pediatric practice by the hospital pharmacy (Table 6). Routinely, pharmacists receive instructions from pediatricians to prepare tailor-made formulations for hospitalized children to meet the following needs:

- Dose adjustment according to patient’s age, weight, creatinine clearance and symptoms;
- change in the dosage form due to inability in swallowing capsules or tablets in patients such as neonates, dysphagic patients and patients fed through PEG (percutaneous endoscopic gastrostomy);
- two drug association non commercially available.

**Table 6:** List of oral drugs routinely manipulated in pediatric practice by the G. Gaslini Hospital Pharmacy.

API	Available Dosage Form	Compounding Process	Dispensed Preparation
Omeprazole	20 mg capsules	Opened and dispersed in a liquid vehicle A proportion of the liquid given	Suspension 2 mg/mL
Mycophenolate mofetil	250 mg capsules		Syrup 100 mg/mL
Tracolimus	5 mg Capsules (Adoport)		Suspension 0.5 mg/mL
Gabapentin	300 mg capsules		Suspension 100 mg/mL
Hydrochlorthiazide	25 mg Capsules (Esidrex)		Suspension 5 mg/mL
Hydrocortisone	10 mg Tablets (Roussel)	Triturated and dispersed in a liquid vehicle A proportion of the liquid given	Suspension 2 mg/mL
Flecainide	100 mg tablets		Suspension 2 or 20 mg/mL
Amlodipine	10 mg tablets (Norvasc)		Suspension 1 mg/mL
Levodopa/carbidopa	100/25 mg tablets (Sinemet)		Suspension 5/1.25 mg/mL
Captopril	Powder Pharm. Eur.	Magistral preparation	Solution 1 mg/mL
Ursodeoxycholic acid	Powder Pharm. Eur.		Suspension 20 mg/mL
Riboflavin	Vitamin B2 Powder Pharm. Eur.		Suspension 10 mg/mL
Phenytoin sodium	100 mg dintoina tablets	Triturated and a portion of the powder given	Chartulae
Bosentan	32 or 125 mg tablets		Chartulae
Indomethacin	50 or 25 mg capsules (Indoxen)	Opened and a portion of the powder given	Chartulae

**Figure 7:**
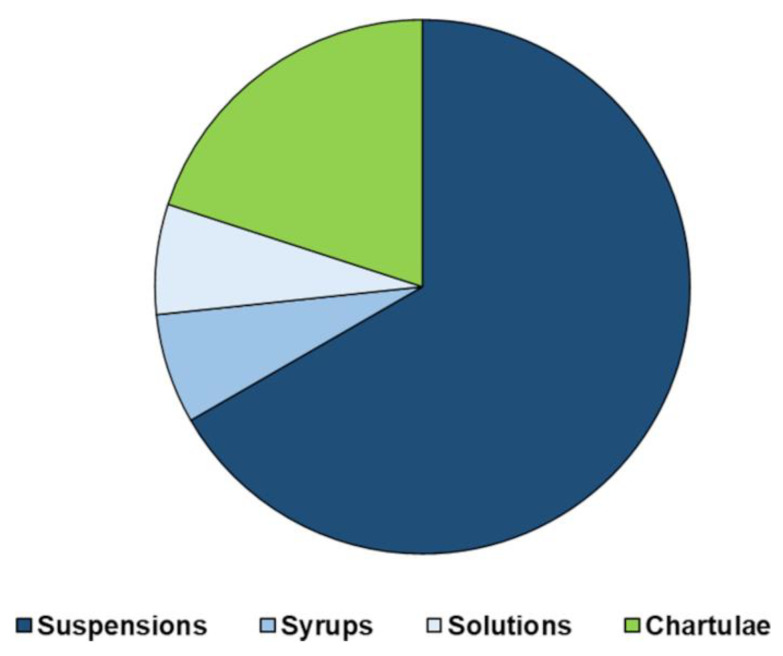
Pediatric preparations developed and dispensed by the G. Gaslini Hospital Pharmacy.

As reported in Table 6, and evidenced by the graph in Figure 7, it is evident that despite the recent advantages of solid dosage forms and their proven suitability for infants, extemporaneous preparations liquids still represent the most common dosage forms in the clinical practice. In the pharmacy practice, flavored sweetened orally suspending vehicles are used to simplify the process involved in the extemporaneous compounding of oral suspensions. Among others, simple syrup, Ora-Sweet^®^, Ora-Plus^®^, and SyrSpend^®^ SF are often employed as suspending agents and administration aids. All-in-one vehicles such as Ora- Sweet^®^ and Ora-Plus^®^ contain flavorings and methylparaben as a preservative. The SyrSpend^®^ SF vehicle has been formulated with more attention to ingredient selection. It contains purified water, modified food starch, sodium citrate, citric acid, sucralose, and sodium benzoate (<0.1%). However, the list of excipients is dangerously expanded each time a liquid formulation is prepared. To ensure the same flexibility in dosages, but without adding questionable excipients, 3D printed MTs could represent a valid alternative in a compounding pharmacy.

## 7. Conclusions

The development of oral pediatric dosage forms is still challenging despite the support from regulatory authorities. Licensed pediatric medicines are still inadequate to cover the overall treatments that these fragile patients need so the dose adjustments starting from the adult dosage forms are a common and questionable practice far to be abandoned. When designing medicines for children, it is of paramount importance to consider the necessity to tune the dose on a patient’s weight and contemplate the safety of the ingredients. For this purpose, mini-tablets (MTs) and mainly micro-tablets (MicroTs) may represent a valid alternative to liquid formulations for their simpler formula and greater stability. This review has provided an overview of different approaches that may be applied to develop MTs and MicroTs intended for the pediatric population with a focus on excipients and flavors selection. Alongside the conventional method of compression, 3D printing is gaining a lot of interest as it allows to reduce the number of ingredients and to avoid both the mixing of powders and intermediate steps such as granulation. These simplifications make this technique well adaptable to the daily galenic preparations of a hospital pharmacy. The commercial availability of this new technology could be increased by enlarging its application to formulate dosage forms for geriatric patients and adults with swallowing difficulties, as well as to support the formulation of off-patent drugs. To achieve a successful patient-centered therapy, a committed partnership between stakeholders would be essential. Academia, hospital pharmacies, and industries should expand their borders to share their skills, experiences, and needs, thus reducing the adult–child medicine development gap. Finally, this synergy and complementarity between pediatric clinics and pharmacists would also be of great help in achieving an adequate optimization of pharmaceutical formulations during chronic therapies in children suffering from rare diseases. In these specific cases, patients require higher levels of service and support; therefore, the opportunity to develop more adequate and safer pharmaceutical formulations would achieve greater compliance with treatments and better outcomes.

## Figures and Tables

**Table 1:** Tolerance limits of the main excipients as currently proposed by the EuPFI database.

Excipient	Function	Toxicity in Children	Limits
Acesulfame K	Sweetener	N.R.	15 mg/kg bw for adults
α-Cyclodextrin	Complexing agent Solubilizing agent	N.R.	Oral toxicity [PDE 120 mg/kg/day; TH neonate—12] Ocular [safe solution <4%; TH neonate—0.1] Parenteral [PD—0.2 mg/kg/day; TH neonate—0.02]
Aspartame	Sweetener	Rare hypersensitivity reactions Cross-reactivity with sulphonamides	Not in homozygous autosomal recessive phenylketonuria patients In patients without dietary restrictions <5 mg/kg/day ADI ≤ 40 mg/kg bw for adults and children
Benzalkonium chloride	Wetting agent Penetration enhancer Antimicrobial preservative Antiseptic Disinfectant Cationic surfactant Solubilizing agent	N.R	ADI of 0.1 mg/kg bw/day ARfD of 0.1 mg/kg bw
Benzoic acid	Antimicrobial preservative	N.R.	5 mg/kg bw for young children
Benzyl alcohol	Disinfectant Solvent Antimicrobial preservative	Gasping syndrome accumulation of metabolites in blood (metabolic acidosis) and brain (neurotoxicity)	Parenteral, Rectal TH—Zero ^1^ Neonates ^2^ Up to three years old ^3^ 5 mg/kg/day for adults and children aged over 4 weeks
β-Cyclodextrin	Complexing agent Solubilizing agent	N.R.	Oral Tox [PDE 10 mg/kg/day; TH neonate—1] Nasal [safe solutio—1.5%; TH neonate—0.15] Ocular [safe solution ±1%; TH neonate—0.1] Rectal [safe amount—5 mg/kg/day or %; TH neonate—0.5] Dermal [safe amount ±0.1%; TH neonate—0.01]
Boric acid	Antimicrobial preservative Buffering agent	N.R.	0.16 mg/kg bw/day for adults
Butylated hydroxyanisole	Antioxidant Antimicrobial preservative	N.R.	1.25 mg/kg bw/day for children
Butylated hydroxytoluene	Antioxidant	N.R.	0.25 mg/kg bw/day for adults
Butylparaben	Antimicrobial preservative	N.R.	Withdrawn ^4^
Cresol	Antimicrobial preservative	N.R.	TDI of 50 µg/kg bw/day for adults
Azo dyes Quinoline dyes Triphenylmethane dyes Xanthene dyes	Coloring agents	Gastrointestinal intolerance Abdominal pain Vomiting Indigestion Hypersensitivity	To be avoided unless necessary
Edetic acid	Antimicrobial preservative Complexing agent	N.R.	1.9 mg/kg of body weight for adults
Ethanol	Penetration enhancer Solvent Antimicrobial preservative	Acute intoxication in accidental overdose Chronic toxicity in routine use CNS depressant Respiratory/cardiovascular toxicities at high concentrations Long-term effects of low doses under discussion	2.6 g/day for adults Blood ethanol levels should not exceed 1 mg/dL after a single dose containing ethanol (or a dose of 6 mg/kg/day) in children aged 2–6 years
Ethylparaben	Antimicrobial preservative	N.R.	10 mg/kg bw for young children
Fructose	Sweetener	↑ blood glucose concentration Laxative effects at high oral doses	Not in patients with diabetes, hypoglycaemia, hereditary fructose intolerance
γ-Cyclodextrin	Complexing agent Solubilizing agent	N.R.	Oral Tox [PDE 200 mg/kg/day; TH neonate—20] Parenteral [PDE—0.8 mg/kg/day; TH neonate—0.08] Dermal [safe amount ±0.1%; TH neonate—0.01]
Iron oxide	Colorant	N.R.	0.5 mg/kg bw for adults
Lactose	Filler diluent in tablets and capsules	Severe prolonged diarrhoea Dehydration Metabolic acidosis in lactose intolerance	Intake of <3 g may provoke the described symptoms sensitivity to lactose varies in severity
Methacrylic acid/ethylacrylate copolymer	Coating material	Fibrosing colonopathy	N.R.
Methylparaben	Antimicrobial preservative	N.R.	10 mg/kg bw for young children
Polyethylene glycol (PEG)	Solvent Diluent, lubricant (tablet, capsule) Ointment base Coating agent Suppository base Plasticizer		10 mg/kg bw for adults
Polysorbates	Solubilizing agent Wetting agent Dispersing agent Emulsifying agent Suspending agent Nonionic surfactant	Liver and kidney failure	10 mg/kg bw for young children
Polyvynil pirrolidone (PVP)	Solubilizing agent		ADI 0–50 mg/kg/day
Polypropylene glycol (PPG)	Plasticizer Stabilizing agent Antimicrobial preservative Disinfectant Solvent Humectant	Neurotoxic effects (Adolescents, schoolchildren) ↑ Death (Low-weight new-borns, preterm babies) Severe brain damage and life-long handicaps Metabolic acidosis Hyperosmolality and laxative effect (Limited metabolic pathway-alcohol dehydrogenase)	1 mg/kg/day—neonates up to 28 days 50 mg/kg/day from 29 days up to 4 years 500 mg/kg—5 years up to 17 years and adults Not in paediatric dialysis patients
Propylparaben	Antimicrobial preservative	Agonistic activity at hormone receptors	10 mg/kg bw for young children Up to 5 mg/kg/day for children >2 years with mature metabolic capacity Recently deleted from the list of permitted food additives in the EU
Saccharin	Sweetening agent	N.R.	5 mg/kg bw for young children ^5^
Sodium benzoate	Antimicrobial preservative Tablet and capsule lubricant	N.R.	5 mg/kg bw
Sorbic acid	Antimicrobial preservative	N.R.	25 mg/kg bw for young children
Sucralose	Sweetening agent	N.R.	5 mg/kg bw/day
Sucrose	Sweetener	Decrease in dental plaque pH Dissolving tooth enamel Promoting dental caries	To be avoided for patients with hereditary fructose intolerance, diabetes For long-term therapy it should be replaced by sugar-free formulations

^1^ Should not be used in pre-term or full-term neonates unless strictly necessary because of the risk of severe toxicity including abnormal respiration; ^2^ not be given due to their immature metabolism; ^3^ should be carefully evaluated and may best be avoided; ^4^ in view of the adverse effects in male rats, butylparaben should be excluded from the group ADI for the parabens used in food. ^5^ it has been recommended that intake of saccharin by children should be minimized; ↑ = high or increasing; PDE = Permitted Daily Exposure; TH = threshold; ADI = Acceptable Daily Intake; ARfD = Acute Reference Dose; TDI = Tolerable Daily Intake; N.R. = not reported.

**Table 2:** Regulatory aspects concerning the safety of flavors.

	Rules and Thresholds in Flavors	Paediatric Age
Conformity to the European laws	Conformity with Regulation EU 1334/2008 on flavorings and certain food ingredients with flavoring properties for use in and on foods and current implementation [32]	Required for all paediatric subpopulations
	Use of flavoring substances permitted and present in the Union List (Annex I of Regulation 1334/2008) in 2012 with Regulation EU 872/2012 [32]	
	Conformity with Commission Implementing regulation EU 872/2012 adopting the list of flavoring substances provided for by Regulation EC 2232/96, introducing it in Annex I to Regulation EC 1334/2008 [33]	
Contaminants	Conformity with Commission Regulation EU 1881/2006 setting maximum levels for certain contaminants in foodstuffs [34]	
Colorants in the flavor	Absent	
Pesticides	Conformity with Regulation EU 396/2005 on maximum residue levels of pesticides in or on food and feed of plant [35]	
Food allergens	Conformity with Annex II of Regulation EU 1169/2011 [36]	
Ethanol in liquid flavors	Absent	Infants/young children < 3 years
	To be evaluated	Children/adolescents 4–18 years
Carcinogenic mutagenic substances	Absent	Infants/young children < 3 years
	To be evaluated	Children/adolescents 4–18 years
Ethanol in liquid flavors	Absent	Infants/young children < 3 years
	To be evaluated	Children/adolescents 4–18 years
Diacetyl	Absent	Infants/young children < 3 years
	To be evaluated	Children/adolescents 4–18 years
Benzyl alcohol	Absent	Infants/young children < 3 years
	<2.5 mg/kg/day	Children/adolescents 4–18 years
Propylene glycol solvent in flavors	<5 mg/kg/day	<1 month
	<25 mg/kg/day	1–36 months
	<25 mg/kg/day	Children/adolescents 4–18 years

**Table 3:** Main advantages and disadvantages of mini-tablets.

Mini-Tablets Features	
Pros	Cons
↓ dose dumping	Price may be higher depending on production technology
↓ inter-intra individual variability	Requirement of excellent powder flow due to the small dies
Good coating substrate	Coating may rupture by accidentally chewing
↓ Local irritation	Limited drug loading capacity per tablet
↓ Capping tendency	Multiple dosing might be necessary due to the limited drug load per single unit
Manufacturing w/o solvent or heating	Packaging or dosing technology platforms needs to be developed
Fine tuning of release rate	
Dose flexibility	
Allow coexistence of different/incompatible drugs	

↓ = Low, reduced.

**Table 4:** Main commercially available Mini-tablets.

Brand Name and Manufacturer	API	Dosage Form	Ingredients	Target Population	Therapeutic Indication
Creon^®^ (Solvay Pharmaceuticals)	Pancreatic enzymes	MT (Delayed release)	Cetyl alcohol Dimethicone Hypromellose phthalate PEG Triethyl citrate	≥6 ms	Chronic pancreatitis cystic fibrosis
Levetiracetam Desitin^®^ (Desitin Arzneimittel GmbH)	Levetiracetam	2 mm MTs (Stick pack)	Povidone K30 Microcrystalline cellulose (MCC) Silicon dioxide (SiO_2_) Magnesium (Mg) stearate Poly(vinyl alcohol) (PVA) Titanium dioxide (TiO_2_) Macrogol 3350 Talc	≥6 years	Epilepsy
Kalideco^®^ (Vertex)	Ivacaftor	2 mm MTs (Stick pack)	SiO_2_ Croscarmellose sodium (Na) Hypromellose acetate succinate Lactose monohydrate Mg stearate Mannitol Sucralose Na lauryl sulfate	≥6 years	Cystic fibrosis
Lamisil^®^ (Novartis)	Terbinafine HCl	2 mm MTs (Stick pack, capsule)	Basic butylated methacrylate copolymer SiO_2_ Dibutyl sebacate Hypromellose Mg stearate MCC PEG Na lauryl sulfate Na starch glycolate	≥4 years	Antifungal treatment (tinea capitis)
Orfiril^®^ Long (Desitin Arzneimittel GmbH)	Sodium Valproate	MTs (sachet/capsule for extended release)	Calcium (Ca) stearate Ethyl cellulose Colloidal SiO_2_ (methylated) Ammonium methacrylate copolymer (type B) Na dodecylsulphate Polysorbate 80 Oleic acid Dibutyldecandioate	≥10 years	Epilepsy
Pancrease MT^®^ (McNeil)	Pancreatic enzymes	2 mm enteric-coated MTs (capsules for delayed release)	Methacrylic acid ethyl acrylate copolimer Cellulose Crospovidone Mg stearate SiO_2_ Triethyl citrate Talc Polydimethylsiloxane Wax Gelatin Iron oxide (Fe_2_O_3_) Polysorbate 80 Na lauryl sulfate TiO_2_	From infancy	Chronic pancreatitis cystic fibrosis

**Table 5:** Mini-tablets (MTs) and micro-tablets (MicroTs) formulations and their distinctive characteristics.

Manufacturing Technique	Formulation (in % *w/w*)	Features	Ref.
Direct compression	Hydrochlorthiazide 15.5% Mannitol-Crospovidone-Polyvinylacetate dispersion (co-processed) 81%, Na stearyl fumarate 3.5%	Oro-dispersible Ø = 2 mm, Friability < 1%, WT = 3 s MU and DCU acceptable, AVs quite high	[52]
	Hydrochlorthiazide 31%, Isomalt 62% Kollidon^®^ CL 4%, SiO_2_ 2% Mg stearate 1%	Oro-dispersible Ø = 2 mm, Angle of repose = 40° MU and DCU acceptable AVs quite high, DT < 30 s 60% of drug released in 5 min (pH 1.2)	[53]
	Enalapril maleate 1.6%, Isomalt 79%, Kollidon^®^ CL-SF 4% Mg stearate 1%	Oro-dispersible Ø = 2 mm, Angle of repose = 34° MU and DCU acceptable, AVs quite high DT ≈ 30 s, 100% of drug released in 7 min at pH 6.8	[54]
	Enalapril 1%, StarLac^®^ 98%, Mg stearate 1%	Oro-dispersible Hardness 39 N, Friability < 1% Mean DC = 103.6%, WT = 23 s, DT = 28 s	[54]
	Risperidone 10%, Mannitol 46% MCC 34% Croscarmellose Na 10% Aerosil^®^ 200/Aerosil^®^ 300 (1/1, *w/w*) 1% Aspartame 0.5%, Peppermint oil 0.5%	Oro-dispersible Ø = 2 mm, Angle of repose = 29° Friability < 1%, Mean DC 98%, DT = 8 s	[55]
Wet granulation (WG) + compression (rotary press)	Hydrocortisone 17%, MCC 22% Lactose monohydrate 52% HPMC 3% Croscarmellose Na 5%, SiO_2_ 0.3%, Mg stearate 1%	Oro-dispersible Ø = 3 mm, Mean DC 102% Completed dissolution after 10 min	[56]
Direct compression	Loratadine 6.7%, MCC 80%, Corn starch 7.3% Croscarmellose Na 5%, Mg stearate 0.5%, SiO_2_ 0.5%	Oro-dispersible Ø = 3 mm, DT 60 s, Hardness 32 N Friability = 0.4% Good MU, 80% of drug released in 3 min	[58]
	Furosemide 10%, Ludipress^®^ LCE 34% Skimmed milk powder 20%, Kollidon^®^ CL-F 20% Mg stearate 1%, SiO_2_ 1% Optarom^®^ Cherry 14 Sucralose 0.5%	Uncoated/Oro-dispersible Ø = 4 mm, DT = 12 s, Hardness 31 N Friability < 1%, Mean DC 94.4% Completed dissolution in 30 min	[59]
	Furosemide 10%, Lactose monohydrate 45% Emdex^®^ 22%, Kollidon^®^ CL-F 20%, Mg stearate 1% SiO_2_ 1%, Strawberry flavor 1%	Oro-dispersible Ø = 4 mm, DT 128 s, Hardness 19 N Friability < 1%, Mean DC 95.6% Completed dissolution within 30 min	[59]
API cocrystallization + direct compression	Piroxicam 41%, Mannitol 19.5%, CMC 10% Croscarmellose Na 5%, Mg stearate 0.5%	Uncoated/Oro-dispersible Ø = 4 mm, Friability < 1% DT = 1 min, 80% drug released in 6 min	[60]
Direct compression	Lapatinib/HPMCP 1/3 spray dried 20% Croscarmellose Na 6%, CMC 71%, Mg stearate 1% SiO_2_ 2%	Uncoated Ø = 2 mm, Friability < 1%, Hardness 25 N Mean DC 107% DT 15 min, 65% drug released Pharmacokinetic study in juvenile porcine model	[61]
Hot melt extrusion	Ketoprofen 40%, EPO 60%	Coated Ø = 5 mm, Friability < 1%, Mean DC 94–110% Completed dissolution in 20 min	[63]
Compressed electrospun nanofibers	Prednisolone 10%, PVP 90%	Uncoated Ø = 2 mm, Friability < 1%, Mean DC 96% Completed dissolution in 20 min	[64]
FDM printing + HME	Caffeine or Propranolol HCl 10% HPMC or HPC 79.5% PEG 6000 10%, Fumed silica 0.5%	Uncoated Ø = 1.5–2–3–4 mm, Mean DC not determined Passed MU, 80% drug released after 175 min	[69]
FDM printing + HME	Baclofen 10%, PVA 80%, Sorbitol 10%	Uncoated Mini-caplets L/W/H 7.5/4/2.5 mm, Hardness 450 N, Good MU DT > 20 min, 75% drug released in 60 min	[70]
	Nifedipine 50%, HPC 34%, Kollidon^®^ VA 64 10% PEG 4000 5%, Mg stearate 1%	Uncoated channeled Ø = 6 mm, Hardness 11 N, Friability < 1% Mean DC 50% 60% drug released after 4 h (burst release)	[71]
FDM printing via passive diffusion drug loading	Nifedipine 4%, PVA-PEG 96% ^1^	Uncoated Ø = 6 mm, Hardness 403 N, Friability < 1% Mean DC 4% Sustained drug release (6 h)	[71]
FDM printing + HME	Indomethacin 20%, HPMCAS 60%, PEG 6000 20%	Chewable soft dosage form Candy-like shapes, Negligible drug release in the mouth 80% drug released in 1 h (pH 7.4)	[73]
Embedded 3D printing	Ibuprofen/paracetamol ^2^, glycerol, gelatin Locust bean gum	Chewable soft dosage form Lego™-like bricks, 100% drug released in 2 h (pH 7.2)	[74]
Direct compression	Ibuprofen 14–25%, Spray-dried Mannitol/MCC ¼ Crospovidone 2%, SiO_2_ 1%, Na stearyl fumarate 4%	Uncoated/Oro-dispersible Ø = 1.5–2.5 mm, 60–100 µm API particle size (D_6_._3_) Friability < 1% MU and DCU passed, DT = 4–90 s	[76]
High shear wet granulation Dry API added to the powder blend	Intragranular: Ibuprofen 0.67%, MCC 10% Mannitol 81.3% Croscarmellose Na 2%, HPMC 2% Extra granular: Croscarmellose Na 2% Na stearyl fumarate 2%	Uncoated Ø = 1.2–1.5 mm, 0.67% *w/w* API loading 18 µm API particle size (D_90_), MU and DCU passed only for 30 units batch 70% API released in 20 min	[77]
High shear wet granulation API nanosuspension added in the granulation fluid	Intragranular: Ibersartam 0.01–0.5%, MCC 9.5%, Mannitol 81 Crospovidone 4%, HPMC 2% Extragranular: SiO_2_ 0.5%, Na stearyl fumarate 3%	Uncoated Ø = 1.2 mm, 0.01–0.5%*w/w* API loading 380 nm API particle size Good MU and DCU per unit at every API loading 97% API released within 10 min	[78]
Direct compression of an interactive mixture	Na salicylate 1%, Mannitol SD 98%, Mg stearate 1%	Oro-dispersible Ø = 2 mm, 1% *w/w* API loading Good DC uniformity, DT 3 min	[79]

^1^ Commercially available hydro-support filaments; ^2^ flexible amounts; L/W/H = length/width/height; Ø = diameter; WT = wetting time; MU = mass uniformity; DCU = drug content uniformity; AVs = acceptance values; DT = disintegration time; DC = drug content.

## Data Availability

Data sharing not applicable.
